# High Pressure and [Ca^**2+**^] Produce an Inverse Modulation of Synaptic Input Strength and Network Excitability in the Rat Dentate Gyrus

**DOI:** 10.3389/fncel.2016.00211

**Published:** 2016-09-27

**Authors:** Thomas I. Talpalar, Adolfo E. Talpalar

**Affiliations:** ^1^Department of Neurobiology, Care Science and Society, Karolinska InstitutetStockholm, Sweden; ^2^Department of Neuroscience, Karolinska InstitutetStockholm, Sweden; ^3^Department of Physiology, Faculty of Health Sciences, Ben-Gurion University of the NegevBeer-Sheva, Israel

**Keywords:** dentate gyrus, hippocampus, diving physiology, high-pressure neurological syndrome, perforant path

## Abstract

Hyperbaric environments induce the high pressure neurological syndrome (HPNS) characterized by hyperexcitability of the central nervous system (CNS) and memory impairment. Human divers and other animals experience the HPNS at pressures beyond 1.1 MPa. High pressure depresses synaptic transmission and alters its dynamics in various animal models. Medial perforant path (MPP) synapses connecting the medial entorhinal cortex with the hippocampal formation are suppressed by 50% at 10.1MPa. Reduction of synaptic inputs is paradoxically associated with enhanced ability of dentate gyrus (DG)’ granule cells (GCs) to generate spikes at high pressure. This mechanism allows MPP inputs to elicit standard GC outputs at 0.1–25 Hz frequencies under hyperbaric conditions. An increased postsynaptic gain of MPP inputs probably allows diving animals to perform in hyperbaric environments, but makes them vulnerable to high intensity/frequency stimuli producing hyperexcitability. Increasing extracellular Ca^2+^ ([Ca^2+^]_o_) partially reverted pressure-mediated depression of MPP inputs and increased MPP’s low-pass filter properties. We postulated that raising [Ca^2+^]_o_ in addition to increase synaptic inputs may reduce network excitability in the DG potentially improving its function and reducing sensitivity to high intensity and pathologic stimuli. For this matter, we activated the MPP with single and 50 Hz frequency stimuli that simulated physiologic and deleterious conditions, while assessing the GC’s output under various conditions of pressure and [Ca^2+^]_o_. Our results reveal that the pressure and [Ca^2+^]_o_ produce an inverse modulation on synaptic input strength and network excitability. These coincident phenomena suggest a potential general mechanism of networks that adjusts gain as an inverse function of synaptic inputs’ strength. Such mechanism may serve for adaptation to variable pressure and other physiological and pathological conditions and may explain the increased sensitivity to strong sensory stimulation suffered by human deep-divers and cetaceans under hyperbaric conditions.

## Introduction

**Figure 1:**
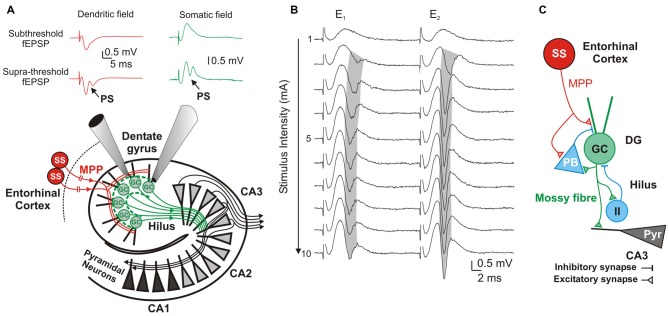
**Components and information flow in the dentate gyrus (DG) network. (A)** Schematic of the network showing information-transfer from spiny stellate (ss) neurons through the medial perforant path (MPP) to the DG and Cornu ammonis 1–3 (CA1–3) fields. Inserts show typical dendritic (red) and somatic (green) recordings at, respectively, the DG’s inner molecular layer and stratum granulossum. Top: Subthreshold fEPSPs; Bottom: Suprathreshold fEPSP eliciting a field granule cell (GC)’s action potential (population spike; PS). **(B)** DG response to paired-pulse stimulation to the MPP with a 20 ms inter-stimulus interval. Progressively stronger stimuli (range 1–10 mA) produce increasingly larger fEPSPs until saturation of the response. Supra-threshold fEPSPs (E_1_ and E_2_) elicit PS_1_ and PS_2_. Note the gradual shortening of PS_1_’s latency (gray shadow) while increasing stimulus intensities, and its prolongation during the strong stimulation (7–10 mA) by probable recruitment of feed-forward inhibition. **(C)** Diagram of the DG circuit including feed-forward inhibition by potential pyramidal basket-cells (PB), and feedback inhibition by unidentified inhibitory interneurons (II) that are activated by GC axons (mossy fibers) at the DG’s hilus.

Pressure is an environmental variable that affects the physiology of living species. Like temperature, pressure produces complex changes in neural network performance (Talpalar and Grossman, 2006; Talpalar, 2007; Marder et al., 2015). Hyperbaric environments, like the depth of the oceans, are inhabited and visited by animals that endure them. The species that inhabit normobaric conditions suffer the high-pressure neurological syndrome (HPNS) when exposed to hyperbaric environments. HPNS is characterized by sensory, motor and cognitive dysfunction, including memory impairment, epilepsy-like episodes and other manifestations of central nervous system (CNS) hyperexcitability, whose severity increases as a function of pressure (Rostain et al., 1983; Bennett and Rostain, 2003; Talpalar, 2007). Professional deep-divers exposed to >1.1 MPa experience HPNS, which impairs their performance and endangers their lives. Toothed-whales spend most of the time at normobaric conditions, but dive for foraging giant squids at depths of 600–2000 m (6.1–20.1 MPa pressure). Diving, turn whales vulnerable to strong stimuli, like navy sonar activity, which apparently leads them to ascend fast, decompression sickness and stranding (Pacini et al., 2011; Tal et al., 2015). We postulated that such vulnerability arises from additive interaction of intense inputs when the CNS operates in “hyperbaric-mode”, a hyperexcitable state that scales up inputs allowing operation at high pressure (Talpalar and Grossman, 2005, 2006). In mammals, spatial orientation (including echolocation) involves the activity of entorhinal inputs onto different fields of the hippocampal formation (Ulanovsky and Moss, 2007; Geva-Sagiv et al., 2015). The medial perforant path (MPP), originating in spiny stellate (ss) neurons of the medial entorhinal cortex (Figure 1A), innervates the proximal dendrites of granule cells (GCs) of the dentate gyrus (DG; Wang and Lambert, 2003). This network serves for normal transfer of context-relevant spatial-orientation cues from the entorhinal cortex to the hippocampus for formation of spatial memory (Leutgeb et al., 2007; Igarashi, 2016). The perforant path is also a gate for cortical epileptic seizures invading the hippocampal formation (Krook-Magnuson et al., 2015) potentially causing amnesia after ictus (Brun et al., 2001). The GCs in the dorsal DG receive information from various areas in the cortex and in particular from the entorhinal cortex (Figure 1A). The MPP conveys a variety of relatively asynchronous and moderately synchronous signals during normal behavior (Sullivan et al., 2011, 2014), and highly synchronous signals when cortical epilepsy propagates to the hippocampal formation (Mody and Heinemann, 1987: Fujita et al., 2014). HPNS displays both, lower performance in cognitive tasks associated with cortico-hippocampal function (Vaernes et al., 1988; Abraini, 1997) and predisposition to epileptic activity (Vaernes et al., 1982; Vaernes and Hammerborg, 1989).

High pressure depresses synaptic transmission by cortical (Talpalar and Grossman, 2003) and hippocampal inputs (Fagni et al., 1987a), which are reduced by about 50% at 10.1 MPa. Synaptic transmission depends on the extracellular calcium concentration ([Ca^2+^]_o_) for triggering the release of synaptic vesicles (Katz and Miledi, 1965; Dodge and Rahamimoff, 1967). Decreasing [Ca^2+^]_o_ reduces MPP’s field excitatory postsynaptic potentials (fEPSPs) simulating high pressure (Talpalar and Grossman, 2003), while increasing [Ca^2+^]_o_ partially reverses pressure-induced synaptic depression of single MPP fEPSPs (Talpalar et al., 2010). Hyperbaric conditions depress synaptic inputs, but increase the ability of GCs to elicit action potentials in response to MPP synaptic stimulation (Talpalar and Grossman, 2004). The DG is known to display low-pass filter properties while transferring cortical activity to the CA3 hippocampal field (Buzsáki et al., 1996). High pressure partially alters such properties increasing the high-pass band of the filter, which can underlie the expression of HPNS in this system (Talpalar and Grossman, 2004). We postulated that increasing [Ca^2+^]_o_ at high pressure, in addition to increase synaptic inputs, may also restore the filter properties of the network and restrain pressure-induced hypersensitivity to strong stimuli. To assess this hypothesis, we studied cortico-hippocampal information transfer of single and frequency responses and evaluated its input-output function under various pressure and [Ca^2+^]_o_ conditions. Pressure and [Ca^2+^]_o_ modulated synaptic release in their expected manner. Strengthening of synaptic inputs was associated to decrease in network gain and increased DG’s low-pass filter properties, while weakening inputs produced the opposite. Those phenomena suggest that global inputs’ strengths may determine the filter properties of a network. Control of network properties by modulation of global inputs’ strengths may be a mechanism of adaptation to physical factors, like temperature and pressure, and a target for neuromodulators that widely affect synaptic release.

## Materials and Methods

### Brain Preparation

Animal experiments were carried out in accordance with the guidelines of the ethics committee for the care and use of animals for experimental work of Ben-Gurion University of the Negev, Beer-Sheva, Israel. Sprague-Dawley rats of both sexes (150–250 g) were euthanized (pentobarbital, 60 mg/kg). Brains were extracted (<1 min) and submerged in cold Ringer’s solution (4–6°C). Cortico-hippocampal slices (400 μm) were prepared as previously described (Talpalar and Grossman, 2003). Brains were sliced with a horizontal vibratome (Campden Instruments) and conserved in an incubation chamber at 25°C for later use. The control Ringer’s solution contained (in mM) 124 NaCl, 3 KCl, 2 CaCl_2_, 2 MgSO_4_H, 1.25 NaH_2_PO_4_, 26 NaHCO_3,_ and 10 D-glucose. The solution was constantly bubbled with 95% O_2_–5% CO_2_ for a pH of 7.4. Variable [Ca^2+^]_o_ was obtained by changing the concentration of CaCl_2_ in the solution.

### Pressure and Compression

High pressure experiments were performed in a pressure chamber (Canty Inc., Lockport, NY, USA). The chamber was provided with an internal experimental bath containing two stimulation electrodes and a temperature gauge. Cortico-hippocampal slices were superfused with pre-warmed Ringer’s solution. The recording pipette was positioned in the DG fields using an electrically remote-controlled manipulator (Talpalar and Grossman, 2003, 2004; Talpalar et al., 2010). Hyperbaric pressure was obtained by compressed helium, a gas that is chemically inert at these pressures (0.1–10.1 MPa). Recordings in some control experiments in the pressure chamber were taken at 0.2–0.4 MPa because these small pressures were more stable than at 0.1 MPa for further pressurization. The rates of compression-decompression ranged between 0.1 and 0.2 MPa/min. Samples were collected at control (0.1–0.4 MPa), 5.1 and 10.1 MPa if not otherwise specified. Control experiments using simultaneous dual recording electrodes in the proximal dendrites and somatic layers of the DG were performed in a conventional setup consisting in a submersion chamber mounted on an air table and similar electronic amplifiers. Ringer’s solution (saturated at normal pressure with 95% O_2_–5% CO_2_) was forced into the experimental bath using a high-pressure pump (LDC analytical minipump). Samples were taken under strict conditions of temperature (30°C) and at least after 15–20 min of stable recording. This time excludes the time needed for stabilization of temperature transients of ±2°C during the processes of compression-decompression (≤32 and 28°C, respectively).

### Electrophysiological Recordings

The conditions of the hyperbaric chamber only allow performing single electrode recordings of extracellular field potentials. Some experiments were performed in a conventional setup provided with double recording electrodes. Field-EPSPs and population spikes (PS) activities were recorded at the somatic and inner dendritic areas of the DG using glass micropipettes (1.5–3 MΩ) filled with Ringer’s solution. The MPP was stimulated using tungsten bipolar electrodes that were placed either at the subiculum or the inner dendritic area of the DG (Talpalar et al., 2010). The stimulation protocol was generated using a Master-8 (AMPI, Jerusalem, Israel) digital pulse-generator and delivered through isolation-units (AMPI, Jerusalem, Israel). The MPP was stimulated with pulses of 40–60 μs duration. The standardized stimulus-intensities (usually 10 steps from 1 to 10 mA intensity) varied between the threshold intensity to elicit a minimal response and supra-maximal stimuli that saturated it.

### Data Recordings and Analysis

MPP fEPSPs were recorded at the inner dendritic and GC regions of the DG and both were analyzed in a similar way. We used fEPSP’s slope as a parameter of synaptic strength because it is more reliable and less contaminated with spike activity than the fEPSP’s amplitude (Talpalar and Grossman, 2004). PSs were used to estimate GCs’ spike firing. We used PS’s amplitude as a parameter for GC output unless otherwise specified. We estimated excitability under the various experimental conditions by assessing the generation of PS in response to MPPs’ fEPSPs stimulation. To generate output/input curves, PSs’ amplitudes were plotted as a function of fEPSPs’ slopes produced by increasing stimulus intensities under control and other experimental conditions. Single fEPSP events were delivered every 10–20 s, and trains of five stimuli at 50 Hz (E_1–5_) were delivered at a rate of 1 train/min. Analysis of these responses involved the measurement and comparison of each individual response (E_n_) in the train and the comparison of the effect of frequency on E_n_ slopes. The data were analyzed using Clampfit 10.2 (Axon Laboratory) and Origin 9.0 (OriginLab) software. Events were normalized with respect to the first event (E_1_) if not otherwise specified. The MPP’s fEPSPs show almost exclusively frequency-dependent depression (FDD) during 50 Hz stimulation-frequency.

### Statistical Analysis of Electrophysiological Experiments

The results are expressed as mean ± SEM if not otherwise specified. Since high pressure experiments were time consuming only one experiment was done with a single animal. Each experiment involved several stimulus intensities, which were carried out in a single slice. Paired Student’s *t*-test was used for comparing parameters taken under control and experimental conditions in the same slice. One-factor and two-factor ANOVA for repetitive measurements was used for comparing parameters (slope, integral, etc.) involving frequency response. Results at each condition were compared with other conditions using Holm-Sidak correction in the Origin 9.0 software (OriginLab). The degree of significance was indicated by the values of *p* (the results were considered statistically different for *p* < 0.05).

## Results

### Increasing [Ca^**2+**^]_o_ Enhances Synaptic Input and Reduces DG Excitability Counteracting High Pressure Effects in Single Responses

#### GCs Readiness to Fire at High Pressure Is Determined by the Resultant of MPP-Inputs Suppression and Network-Excitability Enhancement

High pressure slows the kinetics of a single MPP’s fEPSP onto GC of the DG. The fEPSPs’ slope was reduced by about 50% at 10.1 MPa (Talpalar and Grossman, 2003). Despite of fEPSP suppression, the postsynaptic neurons generated PSs that were indistinguishable from controls (Talpalar and Grossman, 2004). Such hyperexcitability suggests a postsynaptic boost that is associated to reduction of synaptic inputs. Increasing [Ca^2+^]_o_ at 10.1 MPa pressure partially reverted pressure-suppression of fEPSPs and saturated the response at a sub-normal level (Talpalar et al., 2010). High [Ca^2+^]_o_ enhances single fEPSP inputs to GCs, but it may increase or decrease PS-generation depending on how it influences the activity of other synapses that determine the DG’s network excitability (Figures 1A,B). To answer this question, we stimulated the MPP with a wide range of intensities, producing unsaturated and saturated fEPSP responses, and evaluated the PS that they generated under various conditions of pressure and [Ca^2+^]_o_.

**Figure 2:**
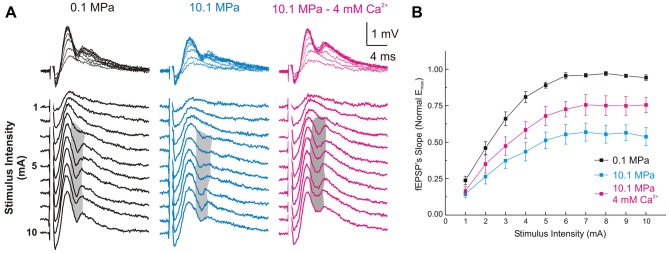
**High [Ca^2+^]_o_ antagonizes high-pressure-induced depression of MPP fEPSPs onto DG’s GCs. (A)** Somatic field recordings at the GC layer of the DG showing fEPSP and PS responses to increasing electric stimulation to the perforant path. **(B)** The MPP fEPSPs’ slopes elicited by all the stimulus intensities are suppressed at 10.1 MPa. Increasing extracellular Ca^2+^ ([Ca^2+^]_o_) from 2 to 4 mM in the Ringer’s solution antagonizes the effect of pressure rising the fEPSPs’ slope at all stimulus intensities (means ± SEM, *n* = 49).

**Figure 3:**
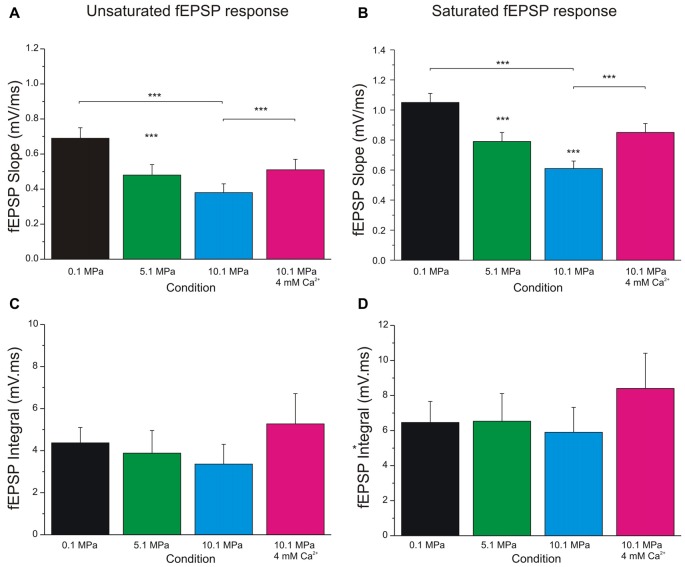
**Increasing extracellular calcium alleviates high pressure depression of MPP fEPSPs.** High pressure effect and the antagonistic effect of increasing [Ca^2+^]_o_ from 2 to 4 mM on MPP fEPSPs. **(A)** Quantification of pressure and [Ca^2+^]_o_ effects on unsaturated fEPSPs’ slopes (*n* = 24 for each condition). **(B)** Effect of pressure and [Ca^2+^]_o_ on saturated fEPSPs’ slopes (*n* = 25 for each condition). **(C)** Quantification of pressure and [Ca^2+^]_o_ effect on unsaturated fEPSPs’ integral (*n* = 5 for each condition; not statistically different). **(D)** Effect of pressure and [Ca^2+^]_o_ on saturated fEPSPs’ integral (*n* = 5 for each condition; not statistically different). Data expressed in mean ± SEM. Significance level, if not otherwise specified, expresses difference with respect to the bar on the left (***p < 0.001).

**Figure 4:**
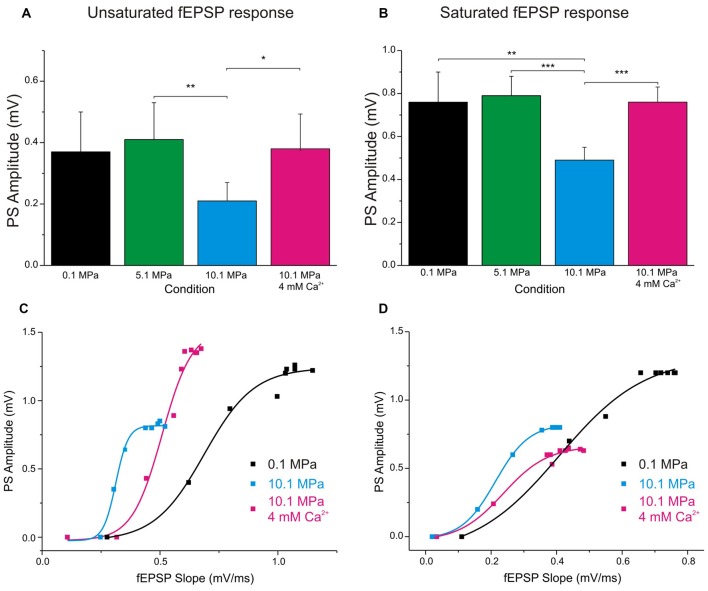
**Raising [Ca^**2+**^]_o_ enhances single fEPSP inputs and reduces DG’s excitability at 10.1 MPa.** Top panels: column bars depict the effect of high pressure and increasing [Ca^**2+**^]_o_ (2 to 4 mM) on the amplitude of PSs elicited by a single fEPSP inputs. **(A)** Effects on the amplitude of PS generated by unsaturated fEPSP inputs (*n* = 20 for each condition). Notice the relatively larger variance for the effect of [Ca^**2+**^]_o_. **(B)** Effects on the amplitude of PS generated by saturated fEPSP inputs (*n* = 25 for each condition). Bottom panels: high pressure increases DG’s excitability in response to depressed fEPSPs while high [Ca^**2+**^]_o_ enhances single fEPSPs but reduces the DG’s excitability. The consequence of these interactions for GCs’ PS-generation depends on which of these antagonistic factors predominates in the system. High [Ca^**2+**^]_o_ often reduces excitability, but its greater strengthening of fEPSP inputs results in increased PS’s amplitude **(C)**. But occasionally, high [Ca^**2+**^]_o_ increases fEPSPs’ inputs while dominantly reduces DG excitability, resulting in decreased PS’s amplitude **(D)** (*p < 0.05, **p < 0.01, ***p < 0.001).

Evaluation of MPP fEPSPs allows the assessment of the status of the DG network input under different conditions. Electrical stimulation of the MPP with progressively stronger intensities activates a larger number of axons producing steeper fEPSPs’ slopes (Talpalar and Grossman, 2003, 2004). Their dynamic range varies from fEPSPs that are barely distinguishable from background noise to fEPSPs whose slopes saturate (do not increase anymore) as a function of stimulus intensity (Figure 1B). Both, unsaturated or saturated fEPSPs can elicit PS. To unify interpretation of dendritic (Talpalar and Grossman, 2003; Talpalar et al., 2010) and somatic signals (Talpalar and Grossman, 2004) we performed experiments with simultaneous recordings in proximal dendrites and somata of roughly same GCs in the DG (Figure 1A). Dendritic recordings displayed typical negative fEPSPs and a fast positive PS deflections (Figure 1A, left insert) while somatic recordings showed an inverse configuration (Figure 1A, right insert) confirming that they can be interpreted similarly. Increasing the stimulus intensity to MPP fibers accelerated fEPSPs’ slopes until reaching saturation (at about 5 mA; Figure 1B). The earliest PS was often generated by an unsaturated fEPSP elicited by 2–5 mA stimulus intensity. PS’s latency was often shortened while increasing stimulus intensity (Figure 1B, gray shadow). The latency for PS’s onset varied from 1.5 to 5 ms after the onset of a saturated fEPSP (*n* = 39). Some experiments showed a mild reduction of PS’s amplitude or increase in PS’s latency during supramaximal stimulation of the MPP (Figure 1B, stimuli 7–10 mA), which suggested partial recruitment of feed-forward inhibition, presumably mediated by pyramidal basket cells (PB; Figure 1C). High pressure reduced fEPSPs’ slopes in a pressure-dependent manner (Figures 2A,B). Unsaturated fEPSP responses (Figures 2A,B; stimuli 1–5 mA; Figure 3A) were reduced by 32 ± 3% at 5.1 MPa (*n* = 24, *p* < 0.001) and by 48 ± 4% at 10.1 MPa with respect to control pressure (*n* = 24; *p* < 0.001). Increasing pressure from 5.1 to 10.1 MPa additionally reduced fEPSPs’ slopes by 22 ± 3% (*n* = 24; *p* < 0.001). Saturated fEPSPs’ slopes (Figures 2A,B; stimuli 6–10; Figure 3B) were reduced by 25 ± 3% at 5.1 MPa (*n* = 25; *p* < 0.001), and by 44 ± 2% at 10.1 MPa, respect to control pressure (*n* = 25, *p* < 0.001). Pressurization to 10.1 MPa reduced fEPSPs’ slopes by 23 ± 13% with respect to 5.1 MPa (*n* = 25; *p* < 0.001). The depression of fEPSPs’ slopes at 0.1 – 5.1 MPa and 5.1 – 10.1 MPa steps was not different. Pressure effects on unsaturated and saturated fEPSPs’ slopes were not different, suggesting that pressure-reduction of synaptic activity is independent of the number of fibers. Despite of the highly significant reduction of fEPSPs’ slopes at high pressure, the integrals of both, unsaturated and saturated fEPSPs, were not different under the various pressure conditions (Figures 3C,D). Previous studies showed that pressure-depressed fEPSP generated PS whose amplitude was conserved at 5.1 MPa and tended to reduction at 10.1 MPa (Talpalar and Grossman, 2004). We revisited this question using a more complete set of MPP stimulus intensities. These data rely exclusively on slices that elicited PSs under at least one experimental condition. PS generated by unsaturated fEPSPs had amplitudes of 0.37 ± 0.13 mV at control pressure, 0.41 ± 0.12 mV at 5.1 MPa (*n* = 20; not significantly different from control), and 0.21 ± 0.06 mV at 10.1 MPa (*n* = 20; not different from control). PS’s amplitudes at 5.1 MPa were significantly larger than at 10.1 MPa (Figure 4A) suggesting that PS generation tends to increase at 5.1 MPa and to decrease at 10.1 MPa. PSs elicited by saturated fEPSPs had amplitudes of 0.76 ± 0.14 mV at normal pressure (*n* = 25), 0.79 ± 0.09 mV at 5.1 MPa (*n* = 25, not statistically different), and were significantly reduced to 0.49 ± 0.06 mV at 10.1 MPa with respect to 0.1 MPa (*n* = 25; *p* < 0.01) and to 5.1 MPa (*n* = 25; *p* < 0.0001). Although all fEPSPs were depressed by high pressure, there was variance in the individual susceptibility of different slices. To correlate individual changes in fEPSPs’ inputs to PS’s amplitudes, we plotted PSs’ amplitudes as a function of fEPSPs’ slopes (Figures 4C,D). The curves showed that subthreshold fEPSPs’ slopes at control-pressure turned supra-threshold under hyperbaric conditions. The output of unsaturated fEPSP inputs showed more variance than those generated by saturated inputs, which were suppressed at 10.1 MPa confirming a previous trend (Talpalar and Grossman, 2004). Altogether, these results show that the DG network copes with reduction of MPP input at high pressure by increasing the gain, but loses control when inputs are too depressed.

### Raising [Ca^**2+**^]_o_ Antagonizes Pressure—Suppression of Single MPP Inputs and Decreases DG Network Excitability

Increasing [Ca^2+^]_o_ from 2 to 4 mM at 10.1 MPa accelerated somatic MPP fEPSPs’ slopes (Figures 2A,B). Unsaturated fEPSP responses increased from 0.38 ± 0.05 to 0.54 ± 0.06 mV/ms (by 55 ± 11%; *n* = 24; *p* < 0.001; Figure 3A) while saturated fEPSPs’ slopes increased from 0.61 ± 0.05 to 0.85 ± 0.06 mV/ms at 4 mM [Ca^2+^]_o_ (by 47 ± 6%; *n* = 25; *p* < 0.001; Figure 3B). Raising [Ca^2+^]_o_ increased unsaturated and saturated fEPSPs’ slopes but kept them 24 ± 4% (*n* = 24; *p* < 0.001) and 19 ± 3% slower than at control pressure (*n* = 25; *p* < 0001). Unsaturated and saturated fEPSPs’ slopes at 10.1 MPa—4 mM [Ca^2+^]_o_ were respectively 13 ± 3% (*n* = 24; *p* < 0.001) and 11 ± 3% (*n* = 25; *p* < 0.001) steeper than at 5.1MPa—2 mM [Ca^2+^]_o_. The effect on unsaturated vs. saturated fEPSPs’ slopes was non-significant (Figures 3A,B). Therefore, increasing [Ca^2+^]_o_ homogeneously relieves high-pressure—depressed fEPSPs’ slopes independently of input fibers involvement. Increasing [Ca^2+^]_o_ at 10.1 MPa rendered fEPSPs’ slopes 12% steeper than at 5.1 MPa (Figures 3A,B) and were expected to elicit larger PSs than at 5.1 MPa. However, PSs’ amplitudes elicited by unsaturated fEPSPs increased from 0.21 ± 0.06 to barely 0.38 ± 0.12 mV (*n* = 25; *p* < 0.05) showing no difference with 5.1 MPa (Figure 4A; *n* = 18). Likewise, PSs’ amplitudes elicited by saturated fEPSPs increased from 0.49 ± 0.06 mV to 0.76 ± 0.07 mV at 4 mM [Ca^2+^]_o_ (*n* = 25; *p* < 0.001), not different from 5.1 MPa (Figure 4B, *n* = 25). The fact that stronger fEPSPs at 10.1 MPa–4 mM [Ca^2+^]_o_ and smaller fEPSPs at 5.1 MPa (Figure 3) elicited similar PSs (Figures 4A,B) suggested that high [Ca^2+^]_o_ may induce two opposite effects for PS generation: enhance fEPSP inputs that promotes it, and decrease excitability that prevents it. Output/input curves obtained by plotting PSs’ amplitudes as a function of fEPSPs’ slopes showed that high pressure increases excitability and that high [Ca^2+^]_o_ reduces it (Figures 4C,D). In conclusion, high [Ca^2+^]_o_ produces steeper fEPSP inputs that are likely to increase PS amplitude, but at the same time decreases DG network excitability, which does the opposite. The consequence of this interaction is often the generation of larger PSs (Figure 4C), but their eventual reduction in a smaller set of experiments (Figure 4D). Such dominance could not be correlated to any perceivable condition in the experiments and may result from diverse integrity of connections in the slices.

### Pressure and [Ca^2+^]_o_ Exert Antagonistic Effects on the Frequency Performance of MPP Inputs and GCs’ Outputs in the DG Network

**Figure 5:**
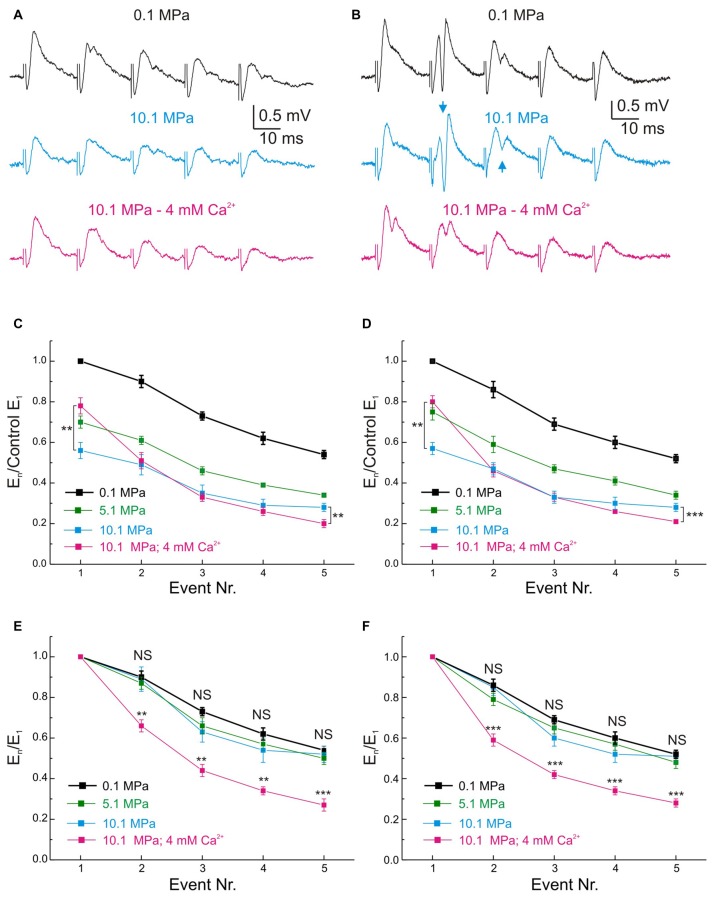
**Effects of pressure and [Ca^**2+**^]_o_ on the MPP fEPSP during stimulation at 50 Hz frequency. (A,B)** Somatic field recordings at the GC layer of the DG showing the performance of fEPSPs under 0.1, 10.1 MPa 2 mM [Ca^2+^]_o_ and 10.1 MPa 4 mM [Ca^2+^]_o_ conditions. **(A)** Effects on subthreshold fEPSPs. **(B)** Effects on saturated suprathreshold fEPSP. Arrows mark the increase of PS strength at high pressure. **(C)** Time course of unsaturated fEPSPs’ slopes at 50 Hz under 0.1, 10.1 MPa 2 mM [Ca^2+^]_o_ and 10.1 MPa 4 mM [Ca^2+^]_o_ conditions (normalized to normobaric E_1_; *n* = 21). **(D)** Course of saturated fEPSPs’ slopes at 50 Hz under 0.1, 10.1 MPa 2 mM [Ca^2+^]_o_ and 10.1 MPa 4 mM [Ca^2+^]_o_ conditions (normalized to normobaric E_1_; *n* = 25). Note that for both fEPSPs’ saturation conditions E_5_ at 4 mM [Ca^2+^]_o_ is significantly weaker than E_5_ at 2 mM [Ca^2+^]_o_. **(E)** Unsaturated fEPSPs’ slopes at 50 Hz under 0.1, 10.1 MPa 2 mM [Ca^2+^]_o_ and 10.1 MPa 4 mM [Ca^2+^]_o_ conditions (normalized to E_1_ at each corresponding condition; *n* = 21). **(F)** Saturated fEPSPs’ slopes during stimulation at 50 Hz under 0.1 and 10.1 MPa—2 mM [Ca^2+^]_o_ and 10.1 MPa—4 mM [Ca^2+^]_o_ conditions (normalized to E_1_ at each corresponding condition; *n* = 25). Notice that in unsaturated and saturated fEPSP responses, high pressure roughly preserves the frequency response of the input at control while high [Ca^2+^]_o_ enhances E_1_ but filters E_2_–E_5_ (**p < 0.01, ***p < 0.001).

**Figure 6:**
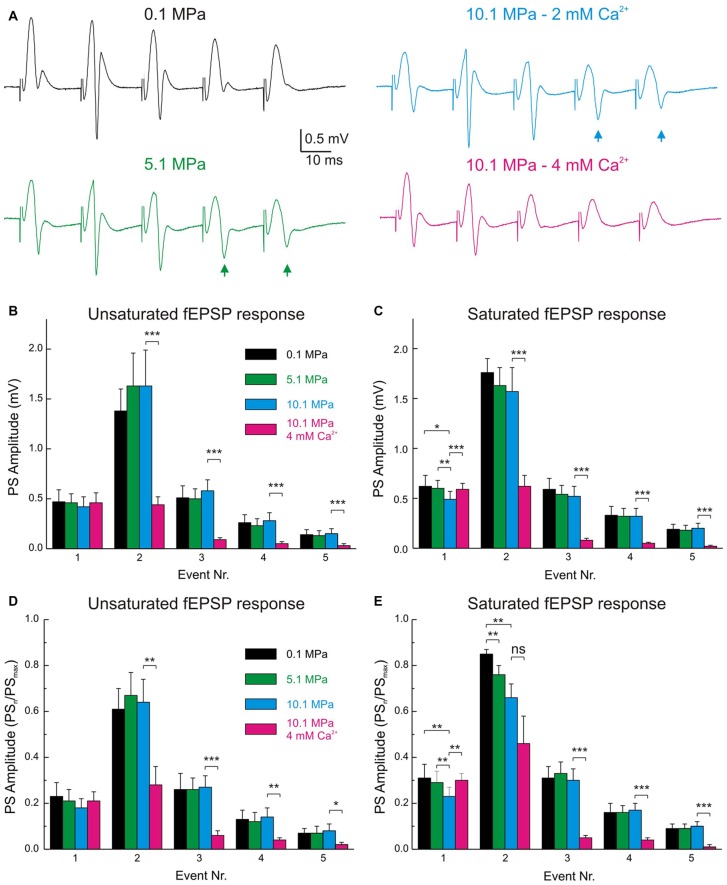
**The band-pass properties of the DG are displaced to high-pass by high pressure and to low-pass by raising [Ca^**2+**^]_o_. (A)** Electrophysiological recordings of typical fEPSPs and PSs during MPP stimulation at 50 Hz under various pressure and [Ca^2+^]_o_ conditions. Note the increase in PS_4–5_ responses at 5.1 and 10.1 MPa (arrows) and their suppression by increasing [Ca^2+^]_o_ from 2 to 4 mM at 10.1 MPa. **(B)** Statistical analysis of the absolute amplitudes of PS_1–5_ elicited by unsaturated fEPSPs at 50 Hz under conditions of variable pressure and Ca^2+^. Individual PS_n_ elicited at the different pressures are not significantly different from their corresponding controls (*n* = 20). **(C)** Course of PS amplitude elicited by saturated fEPSPs at 50 Hz under conditions of variable pressure and Ca^2+^. PSs elicited at the different pressures are not significantly different (*n* = 25). **(D,E)** PS_n_ amplitudes normalized to the maximal attainable PS amplitude (PS_max_) in trains at 50 Hz in any of the conditions. **(D)** PS responses to unsaturated E_n_ at 50 Hz (*n* = 20). **(E)** PS elicited by saturated E_n_ at 50 Hz (*n* = 25). Events were statistically compared with their respective controls using Students’ *t*-test (*p < 0.05; **p < 0.01; ***p < 0.001).

Normal MPP inputs to the GCs in the DG operate at frequencies that include single inputs (≈0.1 Hz), theta-waves (7–12 Hz; Ylinen et al., 1995), gamma-oscillations (40–100 Hz; Bragin et al., 1995) and others. High pressure influences DG network activity by suppressing its single MPP fEPSP input’s strength and modulating its paired-pulse facilitation (PPF) and FDD (Talpalar and Grossman, 2003) while maintaining its output at ≈25 Hz but increasing it at higher frequencies (Talpalar and Grossman, 2004). Such effects imply an alteration of the normal low-pass filter properties of the DG, which may account to its malfunction causing increased sensitivity to sensory stimulation during HPNS (Talpalar and Grossman, 2005, 2006; Talpalar, 2007). High [Ca^2+^]_o_ increased single MPP fEPSPs and accelerated FDD at high-pressure (Talpalar et al., 2010), adding low-pass filter properties to the input. However, it is unclear how high [Ca^2+^]_o_ may affect the frequency response, excitability, and output of the network. Stimulation with trains of five dendritic fEPSPs (E_1_–E_5_) showed that E_1_ and E_3–5_ at 50 Hz were scaled down by about 0.5 at 10.1 MPa (Talpalar and Grossman, 2003; Talpalar et al., 2010). We contemplated the possibility that the pattern observed in dendritic recordings can be altered by the recruitment of voltage-dependent components during its transfer to GC soma. However, somatic fEPSPs elicited at 50 Hz showed that unsaturated (Figures 5C,E) and saturated E_1–5_ (Figures 5D,F) were homogeneously scaled down by a factor of 0.75 at 5.1 MPa and by 0.5 at 10.1 MPa independently of the number of stimulated fibers, and that their FDD was similar to their normobaric controls (Figures 5E,F), displaying a pattern that is similar to dendritic recordings. Increasing [Ca^2+^]_o_ from 2 to 4 mM at 10.1 MPa increased E_1_ by about 50% (overtaking E_1_ at 5.1 MPa by about 12%), but significantly depressing E_2_–E_5_ (Figures 5C,D). We then assessed how high pressure and [Ca^2+^]_o_ modulate the DG output at 50 Hz. Evaluation of GCs’ PS generation by fEPSP inputs at 50 Hz (Figures 5A,B) was carried out in a selected set of experiments to eliminate bias by extremely subthreshold E_1–5_. The criterion for inclusion was that at least one of E_1–5_ elicits a single PS_n_ in the train (PS_1–5_). Figure 6A shows typical somatic fEPSPs and PSs recordings elicited by MPP stimulation at 50 Hz under the various experimental conditions. We compared PS_n_ by calculating their mean absolute amplitude (Figures 6B,C) and their relative strength by normalizing them to the maximal PS’s amplitude (PS_max_) at any condition in each experiment (Figures 6D,E). The means of absolute and normalized amplitudes of PS_1–5_ elicited by unsaturated E_n_ were not different at 5.1 and 10.1 MPa compared to corresponding PS_1–5_ at 0.1 MPa (*n* = 20 for each PS_n_; Figures 6B,D). However, high pressure exerted differential effects on individual PS_n_ generated by saturated E_n_ at 50 Hz. The absolute and normalized amplitude of PS_1_ was conserved at 5.1 MPa (*n* = 25) but both were significantly depressed at 10.1 MPa with respect to 0.1 MPa (*n* = 25; *p* < 0.05) and 5.1 MPa (*n* = 25, *p* < 0.01; Figure 6C), while the normalized amplitudes of PS_2_ at 5.1 and 10.1 MPa were depressed relative to 0.1 MPa (*n* = 25; *p* < 0.01 for each; Figure 6E). In contrast, the mean absolute and relative amplitudes of PS_3–5_ were not different from their respective controls (Figures 6C,E). The sum of PS’s absolute and normalized amplitudes elicited by unsaturated E_n_ was not changed by high pressure. The sums of PS’s amplitudes elicited by saturated E_n_ at 0.1 and 5.1 MPa were similar, but they were reduced at 10.1 MPa (*n* = 25; *p* < 0.05) mostly at the expenses of PS_1–2_. Despite of the conserved or even reduced sum of PS_n_ at high pressure, the number of discernible PS_3–5_ (elicited by E_3–5_) tended to increase at 5.1 and 10.1 MPa (Figure 6A, arrows) confirming that high pressure biases the DG filter response strengthening late PSs in the train (Talpalar and Grossman, 2004). Increasing [Ca^2+^]_o_ from 2 to 4 mM at 10.1 MPa did not change the amplitude of PS_1_ elicited by unsaturated E_1_ (Figures 6B,D) but significantly increased the amplitude PS_1_ elicited by a saturated E_1_ (*n* = 25, *p* < 0.001; Figures 6C,E). Moreover, high [Ca^2+^]_o_ depressed the amplitudes of PS_2–5_ with respect to 10.1 MPa—2 mM [Ca^2+^]_o_ disregarding if they were elicited by unsaturated (*n* = 20; *p* < 0.001 for each PS_n_; Figure 6B) or saturated E_2–5_ (*n* = 25; *p* < 0.001 for each PS_n_; Figures 6C,E). In conclusion, increasing [Ca^2+^]_o_ from 2 to 4 mM at 10.1 MPa enhanced E_1_ while proportionally decreasing E_2–5_, and augmented PS_1_ while weakened PS_2–5_ at 50 Hz disregarding the stimulus intensities. So, these experiments show that high [Ca^2+^]_o_ counteracts high pressure effects on inputs and outputs at 50 Hz and aims to restore the altered filter-properties of the DG. The fact that high [Ca^2+^]_o_ induces larger augmentation on pressure-repressed PS_1_ elicited by saturated E_1_ (Figures 6C,E), or strongly suppresses pressure-enhanced PS_3–5_ (Figure 6), suggests that the effect of [Ca^2+^]_o_ on network parameters is inversely proportional to their sensitivity to pressure. Moreover, they emphasize that the enhancement of low-pass filter properties of MPP inputs by high [Ca^2+^]_o_ is not altered during its convey from the dendrites to the GCs’ somata where PSs are generated.

### Fast GABAergic Inhibition is Essential for High [Ca^2+^]_o_ Restrain of DG Excitability During Single MPP Inputs and Stimulation at Frequency

**Figure 7:**
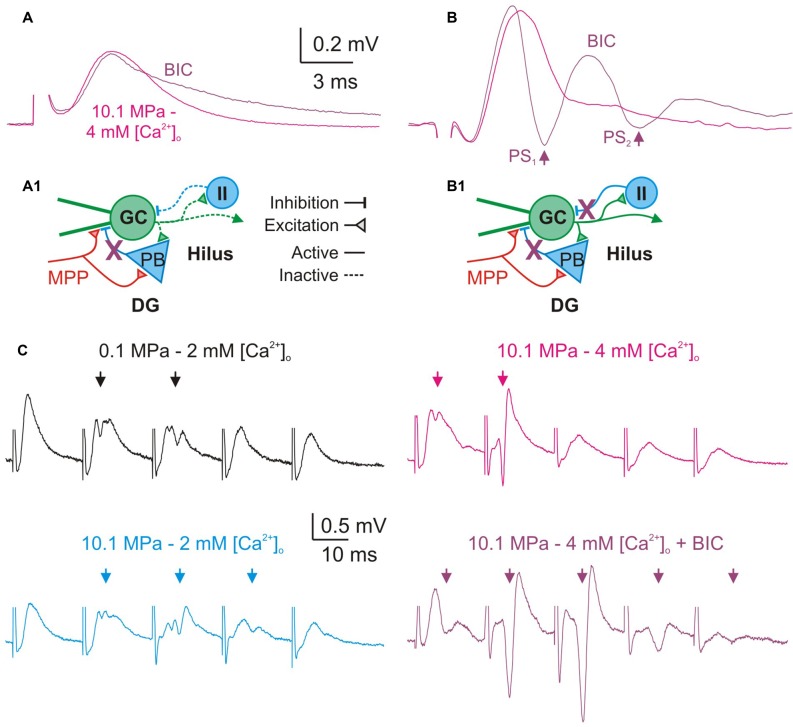
**Blockade of GABA_A_—inhibition increases DG excitability and prevents its restraint by high [Ca^2+^]_o_. (A,B)** Effect of GABA_A_ receptors blockade on single fEPSPs. Bicuculline (BIC, 10–20 μM) prolongs the duration of subthreshold fEPSPs produced by weak MPP stimulation **(A)**, and produces larger PS and repetitive PS firing (arrows) by moderate/intermediate intensity fEPSPs **(B)**. **(A1,B1)** Schematics of a simplified DG network showing basic excitatory and inhibitory elements, suspected targets of GABA_A_ blockade (X), and their consequences for the activity of the network. **(A1)** Blockade of feed-forward inhibition prolongs MPP fEPSPs. During subthreshold MPP fEPSPs stimulation **(A)** the MPP directly excites the GC and the PB producing a monosynaptic fEPSP which is succeeded by a PB’s di-synaptic IPSP which inhibits the late portion of the EPSP. Blockade of inhibition prolongs the duration of subthreshold fEPSPs. **(B1)** Blockade of feed-forward and feedback inhibition produce repetitive firing by supra-threshold MPP fEPSPs. Stronger MPP fEPSP produces a PS that activates feedback inhibitory interneurons (II) suppressing repetitive firing. Thus, blockade of feedback GABA_A_ produces anomalous PS repetitive firing by single fEPSPs. **(C)** GABA_A_ receptors blockade prevents high [Ca^2+^]_o_—mediated restriction of PS_1−5_ generation during trains of moderate/intermediate intensity fEPSPs at 50 Hz. Note the significant increase in the amplitude and number of PS elicited by the E_1−5_ train at 10.1 MPa–4 mM [Ca^2+^]_o_ after blockade of fast inhibition despite of the preserved FDD of the fEPSPs (Arrows).

We postulated that the depressing effect of high [Ca^2+^]_o_ on DG excitability was predominantly mediated by enhancement of feed-forward and feedback GABAergic inhibition. To support this argument we performed a set of experiments that assess how the blockade of fast GABA_A_ inhibition influences the effect of raising [Ca^2+^]_o_ in the DG network. Previous experiments have shown that blockade of GABA_A_ inhibition produced DG hyperexcitability in response to moderate to medium intensity fEPSPs (Talpalar and Grossman, 2004). The present experiments show that blockade of GABA_A_ receptor—mediated inhibition by bath application of bicuculine (BIC, 10–20 μM) did not significantly change fEPSPs’ initial slopes of single fEPSP disregarding stimulus intensity (*n* = 24). We measured the effect of BIC in the amplitude and time course of subthershold fEPSPs. Their amplitude was not significantly changed by BIC (*n* = 10) but the decay time constant was increased by about 31% (*p* < 0.01, *n* = 10; Figure 7A). These results suggests that the duration of MPP fEPSPs in the GC dendrites is limited by feed-forward GABA_A_ inhibition, and therefore its blockade prolongs the decay time of single fEPSPs (Figure 7A1). BIC enhanced the generation of PSs by medium intensity fEPSPs (*n* = 14) promoting the abnormal production of repetitive PS firing by strong fEPSPs (Figure 7B; *n* = 16) eventually producing epileptic-like bouts in the DG (not shown). Such result may arise from the simultaneous suppression of feed-forward and feedback inhibition onto the DG GCs (Figure 7B1). GABA_A_ receptor blockade produces proportionally similar effects at various conditions of pressure (0.1 and 10.1 MPa) and [Ca^2+^]_o_ (2–4 mM) that altogether can be interpreted as letting the excitatory glutamatergic inputs alone to shape the activity of the network.

A previous study showed that during stimulation at frequency, block of GABA_A_ receptors abolished low-pass filter properties of the DG (Talpalar and Grossman, 2004). We have postulated that high [Ca^2+^]_o_ was able to restore the DG’s low-pass filter properties at 10.1 MPa by potentially boosting inhibition in the network (Figure 6). To test this hypothesis we compared the effect of [Ca^2+^]_o_ in preparations proficient and devoid of GABA_A_ inhibition. We assessed the effect of BIC (10–20 μM) on PS generation by trains of moderate—medium intensity fEPSPs at 50 Hz under various conditions of pressure and [Ca^2+^]_o_ (2–4 mM). Blockade of GABA_A_ inhibition eliminated or severely reduced the low-pass filter properties of the DG (*n* = 24). Moreover, blockade of GABA_A_ receptors prevented restoration of these properties by high [Ca^2+^]_o_ (Figure 7C; *n* = 7). Increasing [Ca^2+^]_o_ to 4 mM at 10.1 MPa reduced the average number of discernible PS_1−5_ from 3 to 1.6 (*p* < 0.01), addition of BIC increased the number of PS to 4.14 (*p* < 0.001; *n* = 7). Comparison of E_1−5_ slopes and time course before and after BIC showed indistinguishable MPP FDD at both conditions showing that GABA_A_ inhibition does not influence presynaptic MPP activity, and its blockade preserves its original configuration (*n* = 7).

Bath application of inhibitory blockers caused high intensity single fEPSPs to produce long-lasting bursts of epileptiform repetitive PS activity whose duration overlapped with several of E_1−5_ events at 50 Hz, precluding the analysis of such responses (*n* = 7; not shown). The increase in the decay time of single subthreshold fEPSP attests for blockade of feed-forward inhibition that is presumably recruited by MPP fibers during single fEPSPs (Figures 7A,A1). The hyperexcitability observed on higher intensity fEPSPs and the loss of low-path filter properties of the DG are caused by a combined blockade of feed-forward and feedback GABA_A_ inhibition (Figures 7B,B1,C). In conclusion, these experiments show that intact GABA_A_ inhibition is necessary for high [Ca^2+^]_o_—mediated reduction of excitability at high pressure and support the hypothesis that such effect is mediated by enhancement of fast inhibitory pathways.

## Discussion

The performance of cortico-hippocampal networks is probably disturbed by high pressure producing the cognitive consequences of HPNS seen in humans and other diving animals (Talpalar and Grossman, 2005, 2006; Talpalar, 2007). The *in vitro* cortico-hippocampal network is modulated by high pressure showing depression of MPP inputs to the DG (Talpalar and Grossman, 2003) together with hyperexcitability, and distortion of the DG’s low-pass filter properties (Talpalar and Grossman, 2004). High-pressure depression of single MPP inputs is antagonized by raising [Ca^2+^]_o_, which enhances it at high pressure (Talpalar et al., 2010). Similar effects were seen in vertebrate synapses (Golan and Grossman, 1992), suggesting that synaptic depression by high pressure and synaptic enhancement by raising [Ca^2+^]_o_ are general effects on synaptic activity, independent of the type of synapse. How is this logic applicable to predict a behavior of a network? A simplistic prediction is that increasing MPP inputs under the hyperexcitable hyperbaric conditions will increase the output. We show here that this prediction tends to be correct for single stimuli. However, the normal DG network performance entails activity at different frequencies (Bragin et al., 1995; Ylinen et al., 1995). How increasing synaptic strength may influence the output of a network that contains excitatory and inhibitory synapses? We hypothesized that relieving synaptic suppression at high-pressure may decrease DG network excitability and normalize its frequency response. Our experiments confirm that raising [Ca^2+^]_o_ at high-pressure not only mitigate synaptic suppression, but also reduces DG network excitability in both, single events (Figures 4C,D) and during stimulation at 50 Hz frequency (Figure 6), and therefore restores the pressure-disrupted low-pass filter properties of the DG.

### Effects of Pressure and High [Ca^2+^]_o_ on fEPSPs and PS’s Generation During Single MPP Stimulation and Low Frequency Activity

This study shows that doubling [Ca^2+^]_o_ from 2 to 4 mM at high pressure increases MPP synaptic transmission and reduces excitability of the network. The conclusion is that increasing pressure depresses single MPP inputs, while increasing [Ca^2+^]_o_ enhances them disregarding the number of stimulated fibers or degrees of synchronization. The reasons for such effects were discussed previously (Talpalar and Grossman, 2003), and respond to the dependence on Ca^2+^ to trigger synaptic release (Katz and Miledi, 1965; Dodge and Rahamimoff, 1967) and the high-pressure interference with those processes (Talpalar et al., 2010). The generation of PS by single fEPSPs was conserved at 5.1 MPa and reduced at 10.1 MPa, with a more marked reduction for PSs elicited by saturated fEPSPs, while raising [Ca^2+^]_o_ restored them to control values (Figures 4A,B). Conservation of PS amplitudes was related to hyperexcitability of the DG under hyperbaric conditions (Talpalar and Grossman, 2004), while PS reduction was attributed to extreme suppression of MPP input at 10.1 MPa. High [Ca^2+^]_o_ enhanced the input, but decreased DG network excitability. Their balance often resulted in increased PS output (Figure 4C) or eventual depression of it (Figure 4D). The mechanisms that modulate excitability are complex, and probably involve synaptic and non-synaptic influences. The enhancement of fEPSPs’ slopes by high [Ca^2+^]_o_ in somatic recordings (Figures 3A,B) was larger than in dendritic recordings (Talpalar et al., 2010) suggesting that the former collects converging inputs from many dendrites in the way to the GC somata showing additive effects. Previous studies suggest that synaptic inputs at high pressure are more prone to recruit voltage-dependent post-synaptic components like NMDA receptors (Fagni et al., 1987b; Mor and Grossman, 2007) or voltage-activated currents (Stuart and Sakmann, 1995). Such mechanisms may keep single PS generation despite of fEPSP depression at high pressure. Conserved fEPSPs’ integral (Figures 3C,D) and decay time (Talpalar and Grossman, 2003) at high pressure may be interpreted in that direction. However, if somatic fEPSPs display an integrated response involving synaptic receptor currents together with recruited voltage-activated currents, it is expected that stronger fEPSPs may be less depressed by pressure or more amplified by [Ca^2+^]_o_, but the results were less conclusive (Figure 3). Raising [Ca^2+^]_o_ exerted an indistinguishable effect on unsaturated and saturated fEPSPs slopes (Figures 3A,B), while its tendency to increase fEPSPs’ integrals did not reach statistical significance (Figures 3D,E). Ca^2+^ ions are known to affect many processes, including surface charges on the membrane and ionic currents, which may modulate excitability in the observed trend (Frankenhaeuser and Hodgkin, 1955). But such effects are expected to require larger [Ca^2+^]_o_ fluctuations than in the present experiments. Hyperbaric pressure may increase excitability by general depression of synaptic transmission in the DG network, proportionally affecting its loops depending on the number of synapses that they involve (Talpalar and Grossman, 2006: Talpalar, 2007). Thus, di- or poly-synaptic pathways are more sensitive than monosynaptic inputs. Local feed-forward inhibition by PB cells onto DG dendrites is a di-or tri-synaptic loop (Figure 1C). High [Ca^2+^]_o_ probably strengthens each of these synapses enhancing inhibition. GABA_A_ inhibition counteracts the NMDA-receptor component of the MPP (Staley and Mody, 1992) and its blockade prolonged fEPSPs’ duration in the DG (Talpalar and Grossman, 2004). Blockade of GABA_A_ receptors prolonged subthreshold MPP fEPSPs (Figure 7A) indicating that feed-forward inhibition shapes the time course of MPP fEPSPs. Thus, an MPP enhancement by high [Ca^2+^]_o_ may also enhance PB cell-mediated feed-forward inhibition (Figure 1C) limiting the excitation of MPP inputs to the GCs (Figure 7A1). Moreover, fast feed-forward and feedback GABAergic inhibition (Figures 7A1,B1) seem to normally prevent repetitive PS firing by the GCs upon MPP stimulation since their blockade produced abnormal GC repetitive PS firing (Figure 7B). Under such blockade high [Ca^2+^]_o_ further enhanced MPP fEPSPs strength producing additional PS firing, suggesting that increasing [Ca^2+^]_o_ reduces excitability by enhancing these two types of inhibition.

### Pressure and Ca^2+^ Exert Mutual Antagonistic Effects on DG Network Activity at High Frequency

Neural activity at high frequency is part of the normal repertoire of activities in many systems including the DG (e.g., gamma oscillations). The energy needs of this activity demand enduring metabolism and supplies that are not always attainable at extreme environmental conditions (Talpalar and Grossman, 2006). High pressure scales down MPP inputs and increased the GCs’ PS output at 50 Hz challenging the DG’s low-pass filter properties (Talpalar and Grossman, 2004). Such high-pass switch was attributed to many factors, including primary enhancement of excitatory components (Fagni et al., 1987b; Mor and Grossman, 2007) and reduction of synaptic inhibition (Zinebi et al., 1988; Talpalar and Grossman, 2004, 2006). Raising [Ca^2+^]_o_ increased the low-pass properties of the input (Talpalar et al., 2010) suggesting that it may contribute to restore the DG low-pass filter properties. Nevertheless, since single inputs potentially recruit voltage-dependent components at high pressure it was uncertain if the pattern seen in dendritic inputs will be conserved after convey to the GCs’ somata. This study shows that MPP inputs keep their low-pass properties at the GCs’ somata (Figures 5E,F) and that high [Ca^2+^]_o_ reduces hyperexcitability in the DG, restricting GC firing at high frequency (Figure 6). These effects amount to restoration of the DG’s low-pass filter properties at high pressure. The mechanisms underlying the dynamics of dendritic fEPSP inputs at frequency, their activation and recovery rates, under various conditions were thoroughly discussed previously (Talpalar et al., 2010). The scaling of somatic E_1–5_ provides more precise preservation of control input dynamics at high pressure, indicating that post-synaptic factors may proportionally amplify and smoothen inputs conveyed to GCs’ somata without distorting its presynaptic pattern (Figures 5A,B). Such proportional boosting and smoothing of inputs may be an adaptive mechanism of the network, allowing neuronal communication with metabolically economic low-input signals at high-pressure (Talpalar and Grossman, 2006). Previous studies suggested maintained low frequency response up to 25 Hz and increased response to 50 Hz or more, which was interpreted as a high-pass expansion of the DG’s low-pass filter band (Talpalar and Grossman, 2004). The present experiments show that low-frequency activity (0.1 Hz) may be more filtered at high pressure than previously expected, and that moderately synchronized MPP inputs produce a stable output at 5.1 MPa, but are potentially depressed at 10.1 MPa (Figures 4A,B). It seems that the DG network switched the low-cut properties of the filter rather than expanded its band-pass to higher frequencies. So, the DG network may filter low-frequency synchronized activity in the hippocampal fields at 10.1 MPa (Figures 4C,D). The increased high-frequency band (50 Hz) of the DG at high pressure (Figures 6A,B,D) may enhance gamma oscillations while keeping constant theta rhythm (the physiological meaning of this bias is unclear). Moreover, these data show that the DG still has low-pass filter properties at high pressure, being able to restrain highly synchronized inputs like cortical seizures invading the hippocampus (Dreier and Heinemann, 1991). The fact that fEPSP-elicited outputs were highly pressure-modulated at 50 Hz, while trains of antidromic action potentials elicited at 25–50 Hz were not (Talpalar and Grossman, 2004), suggests that a network rather than a cellular mechanism underlies such effects. Blockade of GABA_A_ inhibition suppressed the DG’s low-pass filter properties at 50 Hz (Talpalar and Grossman, 2004) and prevented the restoration of low-pass filter properties by increasing [Ca^2+^]_o_ (Figure 7C) suggesting that feed-forward and feedback inhibition (Figures 7A1,B1) are targets for these effects. The time course of GABA_A_ inhibition is adequate to control high frequency activity, suggesting that high [Ca^2+^]_o_ enhances DG’s output cut-off at 50 Hz by augmenting feedback inhibition from hilar interneurons (II) onto the GCs’ tri-synaptic circuit (Figure 1C). Raising [Ca^2+^]_o_ from 2 to 4 mM rendered DG’s low-pass filter properties more restrictive than control, suggesting that a smaller [Ca^2+^]_o_ adjustment may be enough to improve HPNS in animal behavior experiments. This study shows that pressure and [Ca^2+^]_o_ control excitability and filter properties of the DG network. They probably bias the excitation/inhibition balance through inversely tuning the strengths of the DG’s synaptic connections. Inverse regulation of excitability by global synaptic strength may be an exclusive property of the DG network determined by its architecture and synaptic characteristics or a general property of networks. Such adjustment of network activity may allow adaptation to variables like pressure and temperature that globally modulate synaptic transmission (Aihara et al., 2001), or be apt for control by mechanisms such as astrocyte-regulation of [Ca^2+^]_o_, like the masticatory network (Morquette et al., 2015).

## Author Contributions

AET designed and made the experiments and partially analyzed them. TIT analyzed experiments, carried out statistics and participated in making figures. AET and TIT wrote the manuscript.

## Conflict of Interest Statement

The authors declare that the research was conducted in the absence of any commercial or financial relationships that could be construed as a potential conflict of interest.

## References

[B1] Abraini J. H. (1997). Inert gas and raised pressure: evidence that motor decrements are due to pressure *per se* and cognitive decrements due to narcotic action. Pflugers. Arch. 433, 788–791. 10.1007/s004240050346 9049171

[B2] Aihara H. Okada Y. Tamaki N. (2001). The effects of cooling and rewarming on the neuronal activity of pyramidal neurons in guinea pig hippocampal slices. Brain Res. 893, 36–45. 10.1016/s0006-8993(00)03285-6 11222990

[B3] Bennett P. B. Rostain J. C. (2003). “The high pressure nervous syndrome,” in Physiology and Medicine of Diving (5th Edn.), eds Brubakk A. O. Neuman T. S. (Philadelphia, PA: Saunders), 323–357.

[B4] Bragin A. Jandó G. Nádasdy Z. Hetke J. Wise K. Buzsáki G. (1995). Gamma (40–100 Hz) oscillation in the hippocampus of the behaving rat. J. Neurosci. 15, 47–60. 7823151 10.1523/JNEUROSCI.15-01-00047.1995 PMC6578273

[B6] Brun V. H. Ytterbo K. Morris R. G. Moser M. B. Moser E. I. (2001). Retrograde amnesia for spatial memory induced by NMDA receptor-mediated long-term potentiation. J. Neurosci. 21, 356–362. 11150353 10.1523/JNEUROSCI.21-01-00356.2001 PMC6762446

[B100] Buzsáki G. Penttonen M. Nádasdy Z. Bragin A. (1996). Pattern and inhibition-dependent invasion of pyramidal cell dendrites by fast spikes in the hippocampus *in vivo*. Proc. Natl. Acad. Sci. U S A 93, 9921–9925. 10.10.1073/pnas.93.18.9921 8790432 PMC38530

[B8] Dodge F. A. Jr. Rahamimoff R. (1967). Co-operative action a calcium ions in transmitter release at the neuromuscular junction. J. Physiol. 193, 419–432. 10.1113/jphysiol.1967.sp008367 6065887 PMC1365607

[B9] Dreier J. P. Heinemann U. (1991). Regional and time dependent variations of low Mg^2+^ - induced epileptiform activity in rat temporal cortex slices. Exp. Brain Res. 87, 581–596. 10.1007/bf00227083 1783028

[B10] Fagni L. Zinebi F. Hugon M. (1987a). Evoked potential changes in rat hippocampal slices under helium pressure. Exp. Brain Res. 65, 513–519. 10.1007/bf00235974 3556479

[B11] Fagni L. Zinebi F. Hugon M. (1987b). Helium pressure potentiates the N-methyl-D-aspartate- and D,L-homocysteate-induced decreases of field potentials in the rat hippocampal slice preparation. Neurosci. Lett. 81, 285–290. 10.1016/0304-3940(87)90397-1 3323951

[B12] Frankenhaeuser B. Hodgkin A. L. (1955). The effect of calcium on the sodium permeability of a giant nerve fibre. J. Physiol. 128:40.1P. 14392634

[B13] Fujita S. Toyoda I. Thamattoor A. K. Buckmaster P. S. (2014). Preictal activity of subicular, CA1 and dentate gyrus principal neurons in the dorsal hippocampus before spontaneous seizures in a rat model of temporal lobe epilepsy. J. Neurosci. 34, 16671–16687. 10.1523/JNEUROSCI.0584-14.2014 25505320 PMC4261094

[B15] Geva-Sagiv M. Las L. Yovel Y. Ulanovsky N. (2015). Spatial cognition in bats and rats: from sensory acquisition to multiscale maps and navigation. Nat. Rev. Neurosci. 16, 94–108. 10.1038/nrn3888 25601780

[B16] Golan H. Grossman Y. (1992). Synaptic transmission at high pressure: effects of [Ca^2+^]o. Comp. Biochem. Physiol. Comp. Physiol. 103, 113–118. 10.1016/0300-9629(92)90249-p 1356688

[B18] Igarashi K. M. (2016). The entorhinal map of space. Brain Res. 1637, 177–187. 10.1016/j.brainres.2015.10.041 26940561

[B19] Katz B. Miledi R. (1965). The effect of calcium on acetylcholine release fro motor nerve terminals. Proc. R. Soc. Lond. B Biol. Sci. 161, 496–503. 10.1098/rspb.1965.0017 14278410

[B20] Krook-Magnuson E. Armstrong C. Bui A. Lew S. Oijala M. Soltesz I. (2015). *In vivo* evaluation of the dentate gate theory in epilepsy. J. Physiol. 593, 2379–2388. 10.1113/JP270056 25752305 PMC4457198

[B21] Leutgeb J. K. Leutgeb S. Moser M. B. Moser E. I. (2007). Pattern separation in the dentate gyrus and CA3 of the hippocampus. Science 315, 961–966. 10.1126/science.1135801 17303747

[B23] Marder E. Haddad S. A. Goeritz M. L. Rosenbaum P. Kispersky T. (2015). How can motor systems retain performance over a wide temperature range? Lessons from the crustacean stomatogastric nervous system. J. Comp. Physiol. A Neuroethol. Sens. Neural Behav. Physiol. 201, 851–856. 10.1007/s00359-014-0975-2 25552317 PMC4552768

[B24] Mody I. Heinemann U. (1987). NMDA receptors of dentate gyrus granule cells participate in synaptic transmission following kindling. Nature 326, 701–704. 10.1038/326701a0 3031511

[B25] Mor A. Grossman Y. (2007). High pressure modulation of NMDA receptor dependent excitability. Eur. J. Neurosci. 25, 2045–2052. 10.1111/j.1460-9568.2007.05479.x 17439491

[B26] Morquette P. Verdier D. Kadala A. Féthière J. Philippe A. G. Robitaille R.. (2015). An astrocyte-dependent mechanism for neuronal rhythmogenesis. Nat. Neurosci. 18, 844–854. 10.1038/nn.4013 25938883

[B28] Pacini A. F. Nachtigall P. E. Quintos C. T. Schofield T. D. Look D. A. Levine G. A.. (2011). Audiogram of a stranded Blainville’s beaked whale (Mesoplodon densirostris) measured using auditory evoked potentials. J. Exp. Biol. 214, 2409–2415. 10.1242/jeb.054338 21697433

[B101] Rostain J. C. Lemaire C. Gardette-Chauffour M. C. Doucet J. Naquet R. (1983). Estimation of human susceptibility to the high-pressure nervous syndrome. J. Appl. Physiol. Respir. Environ. Exerc. Physiol. 54, 1063–1070. 6853282 10.1152/jappl.1983.54.4.1063

[B32] Staley K. J. Mody I. (1992). Shunting of excitatory input to dentate gyrus granule cells by a depolarizing GABA_A_ receptor-mediated postsynaptic conductance. J. Neurophysiol. 68, 197–212. 1381418 10.1152/jn.1992.68.1.197

[B33] Stuart G. Sakmann B. (1995). Amplification of EPSPs by axosomatic sodium channels in neocortical pyramidal neurons. Neuron 15, 1065–1076. 10.1016/0896-6273(95)90095-0 7576650

[B34] Sullivan D. Csicsvari J. Mizuseki K. Montgomery S. Diba K. Buzsáki G. (2011). Relationships between hippocampal sharp waves, ripples and fast gamma oscillation: influence of dentate and entorhinal cortical activity. J. Neurosci. 31, 8605–8616. 10.1523/JNEUROSCI.0294-11.2011 21653864 PMC3134187

[B35] Sullivan D. Mizuseki K. Sorgi A. Buzsáki G. (2014). Comparison of sleep spindles and theta oscillations in the hippocampus. J. Neurosci. 34, 662–674. 10.1523/JNEUROSCI.0552-13.2014 24403164 PMC3870943

[B36] Tal D. Shachar-Bener H. Hershkovitz D. Arieli Y. Shupak A. (2015). Evidence for the initiation of decompression sickness by exposure to intense underwater sound. J. Neurophysiol. 114, 1521–1529. 10.1152/jn.00466.2015 26133802 PMC4561629

[B37] Talpalar A. E. Giugliano M. Grossman Y. (2010). Enduring medial perforant path short-term synaptic depression at high pressure. Front. Cell. Neurosci. 4:128. 10.3389/fncel.2010.00128 21048901 PMC2967425

[B40] Talpalar A. E. Grossman Y. (2003). Modulation of rat corticohippocampal synaptic activity by high pressure and extracellular calcium: single and frequency responses. J. Neurophysiol. 90, 2106–2114. 10.1152/jn.00894.2002 12711708

[B39] Talpalar A. E. Grossman Y. (2004). Enhanced excitability compensates for high-pressure-induced depression of cortical inputs to the hippocampus. J. Neurophysiol. 92, 3309–3319. 10.1152/jn.00178.2004 15254072

[B38] Talpalar A. E. Grossman Y. (2006). CNS manifestations of HPNS: revisited. Undersea Hyperb. Med. 33, 205–210. 16869534

[B41] Talpalar A. E. Grossman Y. (2005). Sonar versus whales: noise may disrupt neural activity in deep-diving cetaceans. Undersea Hyperb. Med. 32, 135–139. 15926306

[B42] Talpalar A. E. (2007). High pressure neurological syndrome. Rev. Neurol. 45, 631–636. 18008270

[B43] Ulanovsky N. Moss C. F. (2007). Hippocampal cellular and network activity in freely moving echolocating bats. Nat. Neurosci. 10, 224–233. 10.1038/nn1829 17220886

[B44] Vaernes R. Bennett P. B. Hammerborg D. Ellertsen B. Peterson R. E. Toonjum S. (1982). Central nervous system reactions during heliox and trimix dives to 31 ATA. Undersea Biomed. Res. 9, 1–14. 7090080

[B46] Vaernes R. J. Bergan T. Warncke M. (1988). HPNS effects among 18 divers during compression to 360 msw on heliox. Undersea Biomed. Res. 15, 241–255. 3212842

[B45] Vaernes R. J. Hammerborg D. (1989). Evoked potential and other CNS reactions during a heliox dive to 360 msw. Aviat. Space Environ. Med. 60, 550–557. 2751585

[B47] Wang X. Lambert N. A. (2003). Membrane properties of identified lateral and medial perforant pathway projection neurons. Neuroscience 117, 485–492. 10.1016/s0306-4522(02)00659-0 12614688

[B48] Ylinen A. Soltész I. Bragin A. Penttonen M. Sik A. Buzsáki G. (1995). Intracellular correlates of hippocampal theta rhythm in identified pyramidal cells, granule cells and basket cells. Hippocampus 5, 78–90. 10.1002/hipo.450050110 7787949

[B49] Zinebi F. Fagni L. Hugon M. (1988). Decrease of recurrent and feed-forward inhibitions under high pressure of helium in rat hippocampal slices. Eur. J. Pharmacol. 153, 191–199. 10.1016/0014-2999(88)90606-1 2903060

